# Effect of yogurt containing polydextrose, *Lactobacillus acidophilus NCFM and Bifidobacterium lactis HN019*: a randomized, double-blind, controlled study in chronic constipation

**DOI:** 10.1186/1475-2891-13-75

**Published:** 2014-07-24

**Authors:** Daniéla Oliveira Magro, Lais Mariana R de Oliveira, Isabela Bernasconi, Marilia de Souza Ruela, Laura Credidio, Irene K Barcelos, Raquel F Leal, Maria de Lourdes Stesuko Ayrizono, João José Fagundes, Leandro de B Teixeira, Arthur C Ouwehand, Claudio S R Coy

**Affiliations:** 1 Department of Surgery, Faculty of Medical Sciences, Campinas State University, Campinas, Brazil; 2 GASTROCENTRO, Campinas State University, Campinas, Brazil; 3 DuPont Nutrition and Health, Campinas, Brazil

**Keywords:** Chronic constipation, *Lactobacillus acidophilus NCFM*, *Bifidobacterium lactis HN019*

## Abstract

**Background:**

Constipation is a frequent complaint and the combination of a prebiotic and probiotics could have a potentially synergic effect on the intestinal transit. The present study therefore aims to investigate the combination of polydextrose (Litesse®), *L. acidophilus* NCFM® and *B. lactis* HN019 in a yogurt on intestinal transit in subjects who suffer from constipation.

**Methods:**

Patients with constipation were randomly divided into two groups, Control Group (CG) and Treatment Group (TG), and had to eat 180 ml of unflavored yogurt every morning for 14 days. Those in the CG received only yogurt, while the TG received yogurt containing polydextrose, *L. acidophilus* NCFM® (ATCC 700396) and *B. lactis* HN019 (AGAL NM97/09513).

**Results:**

Favourable clinical response was assessed since Agachan score had a significant reduction at the end of the study in both groups and tended to be better in the TG. The subjects in the treatment group also had a shorter transit time at the end of the intervention compared to the control group (p = 0.01).

**Conclusion:**

The product containing yogurt with polydextrose, *B.* lactis HN019 and *L. acidophilus* NCFM® significantly shortened colonic transit time after two weeks in the TG compared to CG and may be an option for treatment of constipation.

## Background

Constipation is a frequent complaint and corresponds to various symptoms such as irregular voiding, sensation of incomplete, painful or forceful voiding, hard stools and abdominal discomfort. Incorrect diet can, in part, explain intestinal motility disorders or dyssynergic defecation [1].

The increase in the incidence of intestinal function disturbances is especially present in developed countries, since the industrialization of food provides access to products of greater conservation, which is accompanied by a reduction of fiber content [2]. Furthermore, the population has adopted a sedentary life style, which is associated with an inadequate diet and results in constipation.

Increased consumption of foods rich in fiber, such as, vegetables, fruit, and whole grain products, make up the primary treatment for constipation, besides the recommendation of an adequate ingestion of liquid (basically water) and the practice of physical activity [3]. Studies have shown that food high in fibers, such as, resistant starch, pectin (fruit) and (hemi) cellulose (grains and cereals) promote an improvement of intestinal peristalsis and an increase in fecal weight, all acting as natural laxative [4]. Polydextrose is a polysaccharide that is partially fermented in the large intestine, but is not digested or absorbed by the small intestine, and a substantial part of it is excreted in the stool. It presents the same properties of fibers and promotes a shorter time passing through the intestines and improves the consistency of the stool [5,6]. It also acts as a substrate for beneficial endogenous microbes, allowing an increase in their levels and activity in the intestinal lumen [7,8].

Probiotics have been defined as live microorganisms which when administered in adequate amounts confer a health benefit on the host [9]. They should be viable in the conditions of the gastric lumen and have the capability to adhere to the intestinal epithelium [10]. Selected probiotic strains have been reported to aid in the prevention and treatment of diarrhea caused by rotavirus and/or antibiotic therapy, reduction in the concentration of enzymes which promote cancer, prevention and/or relief of allergic symptoms in children, normalization of bowel movements and stool consistency in individuals who are constipated [11].

To obtain these benefits, as a rule of thumb a dose of at least a billion colony forming units should be consumed daily. A probiotic preparation can consist of a single strain or a combination of strains and can be in association with other biologically active ingredients. Probiotics are often included in dairy products [12], since these foods provide a preferred growth medium for many of these microorganisms.

Currently, there is great interest in functional foods. There are, however, few studies that evaluate their effect on constipation. The use of probiotics hold great promise, and, among the microorganisms evaluated most are of *Bifidobacterium* and *Lactobacillus*. Two randomized studies using products containing *Bifidobacterium lactis* DN-173 010 showed a significant increase in the frequency of evacuation when compared to the control group [13,14]. Also *B. lactis* HN019 has been shown, in a dose depended manner, to shorten intestinal transit in subjects with self reported long transit times [15].

Using *Lactobacillus casei* Shirota, Koebenick *et al*. [16] demonstrated an improvement of the symptoms associated with constipation in comparison to placebo. However, the results when using probiotics with children who are suffering from constipation are inconsistent. Bu *et al*. [17], showed that the use of *Lactobacillus rhamnosus* Lce35 increased evacuation frequency, however, there was no difference when compared to children who took oral laxatives. Two other studies [18,19] showed no evidence of improvement with constipated children using probiotics.

The combination of a prebiotic and probiotics would have a potentially synergic effect on the intestinal transit, as has been shown [20]. The present study therefore aimed to investigate the combination of polydextrose *L. acidophilus* NCFM® and *B. lactis* HN019 in a yogurt on intestinal transit in subjects who suffer from constipation.

## Methods

### Patients

Volunteers were recruited among subjects with chronic constipation. Subjects were of both genders, and between the ages of 18–45 years. In order to evaluate the degree of constipation, the Agachan score [21] and colonic transit time were used. The inclusion Agachan score was from 10 to 20. Subjects with hypothyroidism, antidepressant users, normal colonic transit time (CTT; 24 h or less) as well as higher than 96 hours were excluded.

The subjects recruited were allocated randomly (permuted-block randomization) within two groups, Control Group (CG) and Treatment Group (TG) and consumed 180 ml of unflavored yogurt every morning for 14 days. Those in the CG received only yogurt, while the TG received yogurt containing polydextrose, *L. acidophilus* NCFM® (ATCC 700396) and *B. lactis* HN019 (AGAL NM97/09513). The clinical evaluations for the Agachan score and the colon transit time were done immediately before the beginning of the experiment and at the end. The patients received a card for making daily entries of evacuations and were warned not to take any laxatives, fiber supplements, neither yogurt nor fermented milk. Complementary evaluations were made: body mass index (BMI), food profile/diet, and the recommended liquid consumption of each participant. The local ethical committee approved the project (N°. 454/2008). Registered under ClinicalTrials.gov identifier no. NCT01825434.

### Yogurt preparation

Unflavored yogurt was prepared on one occasion for the whole experiment. For the TG, 3.6 g of polydextrose (in the form of 4 grams of Litesse®, Danisco Brazil, Cotia) *L. acidophilus* NCFM® and *B. lactis* HN019 (Danisco Brazil, Cotia) were included in the yogurt with, at least 10^9^ colony forming units (CFU) per strain per portion. All test products were packaged, labeled and randomized before the study. The study was blinded to investigators and volunteers and the code was secured in a sealed envelope. The presentation was identical as to the consistency, taste, odor, and appearance.

### Protocol of colic transit study

The patients took one capsule a day which contained 24 markers (Sitzmarks^©^) with three different formats for three consecutive days and had an abdominal x-ray the day after ingesting the third capsule. An evaluation with the total of markers was made before; day 0 and at the end of the study; day 14^th^ day, of eating yogurt.

### Statistical analyses

Fisher’s exact test, Mann-Whitney *U* test and *t*-test were used for the statistical analyses when appropriate. The level of significance was considered at 5%.

## Results

The number of patients assessed was 71 and 24 of them were excluded due to: normal CTT (12); Changed mind on participation (10); inadequate tolerance to yogurt (2); and there were no exclusions due to collateral effects. Of the 47 individuals who participated 43 (91.5%) were women. The Control Group (CG) had 21 participants while the Treatment Group (TG) had 26. No significant statistical differences were observed as to gender, age, BMI, frequency of bowel movements per week (BM) and use of laxatives between the two groups (Table 1).

**Table 1:** Baseline characteristics of treatment vs control groups

	**CG**	**TG**	**p-value**
Female	19 (90.5%)	24 (92.3%)	1.0000**
Male	2 (9.5%)	2 (7.7%)	
Age (years)	32.7 ± 7.3	31.5 ± 7.1	0.5773*
BMI (kg/m^2^)	26.8 ± 4.8	28.2 ± 5.9	0.4475*
BM (/week)	7.4 ± 3.5	6.8 ± 2.6	0.9175*
Use of laxatives	4 (19.1%)	6(23.1%)	1.0000**

*Mann-Whitney, **Fisher test.

Samples of the yogurt were analyzed at the end of the study to quantify the amount of polydextrose as well the probiotic content. The amount of polydextrose was not changed at the end of the study (14 days) and the levels of *B. lactis* and *L. acidophilus* were more than 1×10^9^ CFU/portion for each strain.

Favourable clinical response was assessed since Agachan score had a significant reduction at the end of the study in both groups and tended to be better in the TG (Table 2). The number of bowel movements per day remained unchanged for the duration of the study (Table 3).

**Table 2:** Initial and final Agachan´s score per group (mean, standard deviation)

**Group**	**N.**	**Initial**	**Final**
CG	21	14.3 ± 2.4*^§^	8.3 ± 3.5^§^★
TG	26	13.8 ± 2.1*✗	5.3 ± 2.9✗★

CG control Group, TG treatment group.

Comparison of data with the same symbol; *p > 0.05, ^§^p < 0.001, ✗p < 0.001, ★p = 0.005.

**Table 3:** Bowel movements/day (mean, standard deviation)

**Group**	**N.**	**Initial**	**Final**
CG	21	0.7, 0.3*	0.7, 0.3*
TG	26	0.7, 0.3*	0.8, 0.3*

CG control Group, TG treatment group.

Comparison of data with the same symbol; * p > 0.05.

The subjects in the treatment group also had a shorter transit time at the end of the intervention compared to the control group (*t*-test, p = 0.01), Table 4.

**Table 4:** Colonic transit time (hours) as evaluated by abdominal X-ray of consumed radio-opaque markers

**Group**	**N.**	**Initial**	**Final**
CG	21	37.8 ± 15.2	33.9 ± 12.4^§^
TG	26	35.0 ± 15.4*	24.6 ± 12.8*^§^

Comparison of data with the same symbol; *p = 0.001, ^§^p = 0.01.

## Discussion

Despite constipation being a frequent complaint in specialized clinics, fortunately, only a few of the cases are considered grave by having long periods without evacuating or not responding to the use of laxatives. These are difficult to deal with, making it necessary to perform specific exams, such as, defecography or CTT. In the majority of the cases, the patients present problems associated with bad dietary habits and sedentary life style. In this situation, dietary advice and more frequent use of fibers and sufficient intake of liquids, make it a manageable condition [22], nevertheless with varied results, hence the necessity to search for more efficient treatments. Functional foods could be promising in alleviating motility problems of the gastrointestinal tract, in relation to irritable bowel syndrome and constipation [23]. Besides this, there is data showing that the intestinal microbiota is diverse among healthy individuals and sufferers of constipation [24,25]. In such case, intervention with probiotics could be accompanied by modification of intestinal transit time as observed by Marteau *et al*. [26]. The decrease of the intestinal pH [27], a competitive action with intestinal pathogen(s) or production of substances with neurotransmitter activities [28] are some of the mechanisms attributed to selected strains of probiotics which stimulate the intestinal peristalsis.

In the present study, a reduction of CTT using radio opaque pellets was observed. This reduction was only significant for the Treatment Group and was also significantly different from the final CTT of the Control Group. Although the main inclusion criterion was slow intestinal transit, some volunteers exhibited a normal transit time of around 24 h. Despite this, a significant reduction in transit time was observed for the whole Treatment Group. However, subjects with a close to normal transit time were not suffering from unnecessary further shortening and potential diarrhea. Despite this difference in transit time between the groups, the Agachan score improved in both test groups and was not different between the groups. The used *B. lactis* strain, HN019, has recently been shown to reduce intestinal transit in a placebo controlled dose ranging study [15,29]. The present study confirms these observations. Also other probiotics have been reported to shorten colonic transit; Yang *et al*. [13] saw that the use of *B. lactis* DN-173 010 for periods of over two weeks was accompanied by an increase in the frequency of bowel movements in women who suffered from constipation. A study by Guyonet and co-workers [14] showed an improvement in bowel function in constipated subjects with Irritable Bowel Syndrome after the use of the same *B. lactis* for six weeks. In a similar manner, Koebnick *et al*. [16] verified that the use of *L. acidophilus* Shirota was accompanied by an improvement of the symptoms related to constipation.

The third biologically active component in the test yogurt; polydextrose has also been reported to positively influence stool consistency and bowel function [30] Likewise, Hengst *et al*. [6] reported that a yogurt containing polydextrose reduced oro-faecal transit time, as well as an improvement in the fecal consistency.

## Conclusions

The product containing yogurt with polydextrose, *B.* lactis HN019 and *L. acidophilus* NCFM® significantly shortened colonic transit time after two weeks and may be an option for treatment of constipation.

## Abbreviations

CG: Control Group; TG: Treatment Group; CTT: Colonic transit time; BMI: Body mass index; CFU: Colony forming units; BM: Bowel movements per week.

## Competing interest

The authors declare that they have no competing interests. Daniéla Oliveira Magro, Lais Mariana R. de Oliveira, Isabela Bernasconi, Marilia de Souza Ruela, Laura Credidio, Irene K. Barcelos, Raquel F. Leal, Maria de Lourdes Setsuko Ayrizono, João José Fagundes, Claudio S. R. Coy, Authors with competing interests, Leandro de B. Teixeira and Arthur C. Ouwehand are DuPont Nutrition and Health employees. The authors declare that this article is original and have not been published or submitted to any other scientific publication.

## Authors’ contributions

DOM: design, data analysis and interpretation, contribution to discussion. LMRO: data collection. IB: data collection. MSR: data collection. LC: data collection. IKB: design, data collection. RFL: data collection. MLSA: data collection. JJF: data collection. LBT: design, contribution to discussion. ACO: design, contribution to discussion. CSRC: design, data analysis and interpretation, contribution to discussion, review/edit of manuscript. All authors read and approved the final manuscript.
